# Low Molecular Weight Fucoidan Inhibits Pulmonary Fibrosis In Vivo and In Vitro via Antioxidant Activity

**DOI:** 10.1155/2022/7038834

**Published:** 2022-03-02

**Authors:** Huidan Dong, Tao Xue, Yanjuan Liu, Shan He, Yanliang Yi, Bo Zhang, Jie Xin, Zhen Wang, Xinpeng Li

**Affiliations:** ^1^College of Pharmacy, Linyi University, Linyi, Shandong, China; ^2^Chinese Academy of Traditional Chinese Medicine, China

## Abstract

In this study, sulfated polysaccharides extracted from *Laminaria japonica* were degraded by free radicals to obtain low molecular weight fucoidan (LMWF). The *in vivo* and *in vitro* effects of LMWF on bleomycin-treated pulmonary fibrosis mice and TGF-treated A549 cells, respectively, were evaluated, and the role of antioxidant activity was assessed. H&E, Masson's trichrome, and Sirius red staining results showed that bleomycin induced obvious pathological changes and collagen deposition in the lung tissue of mice. However, LMWF effectively inhibited collagen deposition, and based on immunohistochemistry analyses, LMWF can also inhibit the expression of fibrosis markers. At the same time, LMWF could regulate related antioxidant factors in the lung tissue of pulmonary fibrosis mice and reduce the pressure of oxidative stress. Moreover, LMWF could improve the morphology of cells induced with TGF, which confirmed that LMWF could inhibit fibrosis via antioxidant activity modulation.

## 1. Introduction

Pulmonary fibrosis (PF) is a common clinical, chronic, progressive, and irreversible interstitial lung disease that is characterized by extensive interstitial cell proliferation and extracellular matrix (ECM) deposition [1, 2]. After diagnosis, the 5-year average survival rate of PF patients is only 20–30%, which is lower than survival rates of some types of cancer [2]. Few drugs are currently available for the treatment of interstitial lung diseases, and the most common ones are pirfenidone and Nintedanib [3, 4]. Based on clinical evaluations, these drugs can effectively inhibit idiopathic pulmonary fibrosis, but they have several potential side effects, including gastrointestinal reactions, liver injury, and bleeding. Moreover, the efficiency of pirfenidone and Nintedanib in treating various types of PF remains unknown [5]. Considering that PF is a serious problem affecting public health, there is a great need for the development of safe and effective drugs that can be used in the treatment of PF diseases [6, 7].

Previous studies have confirmed that the occurrence and development of PF are related to the epithelial-to-mesenchymal transition (EMT) of alveolar type 2 epithelial cells (AEC IIs) [8, 9]. Through EMT, pulmonary epithelial cells transdifferentiate into myofibroblasts, which in turn secrete ECM components, thereby triggering fibrosis. As a result of a detailed study, our lab found that oxidative stress is the main inducer of pulmonary fibrosis [10, 11]. The occurrence of PF is closely related to disorders of redox balance caused by the accumulation of reactive oxygen species (ROS) [12]. Compared with healthy people, oxidative stress markers such as H_2_O_2_ and 8-isoprostaglandin F2*α* (8-iso-PGF2*α*) can be found in exhaled respiratory condensate from PF patients. In addition, the content of 8-isopropanolane and oxidized protein in the bronchoalveolar lavage fluid (BALF) of PF patients increases 5-fold and 2-fold, respectively [13–15]. Moreover, the level of glutathione in the epithelial lining fluid and sputum of PF patients was shown to decrease fourfolds compared to healthy patients, indicating that the antioxidant defense of PF patients decreased significantly. In contrast to this, oxidative stress and transforming growth factor (TGF-*β*) have all been shown to significantly promote PF. Persistent lung injury can produce ROS, which can cause the apoptosis of alveolar epithelial cells, damage of basement membrane, and transformation of stroma into epithelium, thus destroying lung structure and damaging alveolar gas exchange. ROS produced by alveolar type II cell injury can cause oxidative stress responses, which can not only induce epithelial cell apoptosis but also activate intracellular signal pathways, upregulating the synthesis and release of fibrosis factors, and finally leading to lung injury and fibrosis. At the same time, intracellular signals triggered by oxidative stress can stimulate fiber proliferation and the expression of fibrosis factors and accelerate the development of PF. Therefore, in the process of PF development, more attention should be paid to the regulation of antioxidant stress, which is also an effective method to inhibit the development of PF.

Sulfated polysaccharides have great potential as active substances in medicine. As it has been long known that sulfated polysaccharides have many pharmacological activities, many studies have reported findings with these compounds in recent years. Sulfated polysaccharides have been shown to kill malaria parasites [16], inhibit the formation of mouse osteoclasts, and participate in the regulation of angiogenesis [17, 18]. In addition, sulfated polysaccharides have been demonstrated to have antioxidant and anti-inflammatory properties [19]. Algae, including *Laminaria japonica*, are readily available marine resources that have great medicinal value and are rich in sulfated polysaccharides. One in particular is low molecular weight fucoidan (LMWF) (molecular weight = 8–10 kd) that can be isolated from *Laminaria japonica*. It is mainly composed of fucose and has antioxidant, anti-inflammatory, and antifibrosis properties [20, 21]. According to previous studies, sulfated polysaccharides extracted from *Laminaria japonica* can effectively reduce bleomycin-induced collagen deposition in mouse lungs, significantly inhibiting the expression of TNF–*α* and vascular endothelial growth factor (VEGF) and improving the symptoms of PF [22, 23]. In addition, the oral administration of fucoidan can delay radiation-induced PF by changing the expression of inflammatory factors in the lung [24]. Despite the well-established effect of LMWF on PF, the mechanisms underlying these effects remain unclear.

This study is an extension of a previous study wherein we show that LMWF can effectively inhibit bleomycin-induced PF in mice. Herein, we investigate the role of antioxidant activity in PF treatment using LMWF, and we analyze the mechanisms implicated in PF pathogenesis and in the therapeutic effect of LMWF.

## 2. Materials and Methods

### 2.1. Reagents and Animals

Bleomycin hydrochloride and monosaccharides were purchased from Hanhui Pharmaceutical Co., Ltd. (Hangzhou, China), Beyotime (Shanghai, China), and Sigma-Aldrich (St. Louis, USA), respectively. All of the other chemicals and reagents were obtained from general commercial sources and used without prior treatment, unless otherwise specified. Antibodies (Collagen, Fibronectin, *α*-SMA, and TGF-*β*) were produced by Affbiotech (Cincinnati, OH, USA), and when used in Western blot or immunohistochemistry (IHC), they were diluted one thousand or one hundred times, respectively, using a primary antidiluent (Beyotime, Shanghai, China). Antibodies for immunofluorescence (Nrf-2, HO-1, and NQO1) were produced by ABclonal (Woburn, USA). Secondary antibodies, including Biotin-conjugated AffiniPure Goat Anti-Mouse IgG (H+L) and Biotin-conjugated AffiniPure Goat Anti-Rabbit IgG (H+L), were provided by Solarbio (Beijing, China). A hydroxyproline ELISA kit was obtained from Bioswamp (Wuhan, China). MDA, GSH, and SOD kits were produced by Solarbio.

All of the experiments were performed on male C57BL/6 mice (22 ± 2 g, SPFII Certificate) bought from Zhong chu Heng tong Biotechnology Co., Ltd. (Jinan, China). All of the experimental procedures performed in this study were previously approved by the Animal Ethics Committee of the School of Pharmacy at Linyi University. Experiments were also performed in accordance with the ARRIVE guidelines (the ARRIVE guidelines 2.0: updated guidelines for reporting animal research). All of the methods were performed in accordance with the relevant guidelines and regulations. Best efforts were made to minimize the suffering of animals.

### 2.2. Chemical Composition of LMWF

LMWF polysaccharides were obtained by water extraction and alcohol precipitation, as described previously [20, 21], and their content was determined using the phenol-sulfuric acid method, using L-fucose as a standard [25]. The content of uronic acid, sulfate, and L-fucose was assessed based on the carbazole colorimetry method, the gelatin-barium chloride method, and the L-cysteine hydrochloride method, using D-glucuronic acid, potassium sulfate, and L-fucose as standards, respectively [26–28]. A high-performance gel permeation chromatography (HPGPC) system equipped with a TSK-G3000 column (300 mm × 7.8 mm) and a refractive index detector was used to determine the molecular weights of the sulfated polysaccharide components. The composition of neutral sugar (monosaccharide) was estimated by high-performance liquid chromatography (HPLC) [29].

### 2.3. In Vivo Experimental Model and Grouping

Male C57BL/6 mice were randomly divided into six groups, namely, Sham (Sham), bleomycin-induced (3.5 mg/kg) PF (PF), bleomycin-induced PF+prednisone (PED) (PF+PED), bleomycin-induced PF+low-dose LMWF (25 mg/kg, PF+L), bleomycin-induced PF+medium dose (50 mg/kg, PF+M), and bleomycin-induced PF+high-dose LMWF (100 mg/kg, PF+H) groups, with 10 mice in each group. All of the mice were subjected to light/dark cycles (12 : 12 h) for one week before experimentation, and they were kept on the same diet throughout the experiment. On the second week, 3.5 mg of bleomycin was dissolved in 1 mL of normal saline, and the injection volume was 1 mL/kg/time. The skin, muscle, and trachea of the neck were cut in the mock group, while in the other groups, the skin, muscle, and trachea of the neck were cut and bleomycin was injected into the trachea. Bleomycin was injected into all of the mice, except the Sham group mice, which were injected with saline instead. The mice were administered with LMWF by intraperitoneal injection for 28 days, while the Sham and PF mice were given saline by intraperitoneal injection.

### 2.4. Assessment of Lung Fibrosis

At the end of the experiment, all of the animals were euthanized (pentobarbital, 30 mg/kg), the lung tissues of the mice were removed and immediately fixed in 4% formaldehyde. After routine paraffin embedding, the tissue samples were cut into 5 *μ*m sections, stained with Masson, H&E, and Sirius red in order to observe collagen deposition and fibrosis pathological changes. Fibrosis scoring was performed according to the established protocol by Ashcroft. The BALF from each animal was centrifuged at 1000 × g and 4°C; then, the supernatant was taken to test hydroxyproline levels. The content of hydroxyproline in the BALF and lung was determined using an ELISA kit. The dry-wet ratio of lung tissue was measured as well. After these experiments, the lower lobe of the right lung of mice was weighed, recorded as wet weight, and then dried at 80°C for 24 hours to a constant weight. This was then weighed and recorded as the dry weight.

### 2.5. Oxidative Stress in the Lung

The content of GSH, MDA, and SOD in lung tissue was determined using kits, the content of hydrogen peroxide in lung tissue was determined using an enzyme biosensor, and the content of 8-isoprostaglandin 2*α* (8-iso-PGF2*α*), heme oxygenase-1 (HO-1), and NAD(P)H: quinone oxidoreductase 1 (NQO1) were detected by ELISA.

### 2.6. Immunohistochemistry and Immunofluorescence Staining

Paraffin sections of lung tissue were used for immunohistochemistry and immunofluorescence analysis. The expression and localization of collagen, fibronectin, *α*-SMA, and TGF-*β* in lung tissue were determined, and the positive area in each section was counted.

### 2.7. Western Blot Analysis

To quantify the proteins in mouse lungs, Western blot analyses were conducted, as detailed in a previous study [30]. First, each sample of lung tissue (20 mg) was treated with 200 *μ*L of lysis buffer. Subsequently, the resulting mixture was centrifuged at 4°C and 12,000 × g for 10 min. To determine the total protein concentration, the supernatant was collected and analyzed using a BCA Protein Assay kit (the measurement process was conducted on ice). After denaturation, Western blot analyses were conducted in order to visualize proteins. The results were then analyzed by chemiluminescence, using *β*-actin as a loading control.

### 2.8. In Vitro Experiments

A549 cells (human lung cancer cell line) were grown in Ham's F-12K medium supplemented with 20% fetal bovine serum (Gibco, Grand Island, NY, USA), 2 mM L-glutamine, 100 U/mL penicillin, and 100 *μ*g/mL streptomycin. These cells were cultured to subconfluence at 37°C in a water-saturated atmosphere composed of 5% CO_2_. When the cells reached 90% confluency, the cells were transferred to 96-well plates, with 2 × 10^3^ cells in each well. Following 24 h starvation in serum-free medium, the cells were treated with LMWF (10–640 *μ*g mL^−1^) and cultured for 72 h in six-well plates.

The cells were then divided into six groups, namely, the normal (NG), TGF-*β*1-induced (10 ng mL^−1^ TGF-*β*1), TGF-*β*1-induced+PED (PED), TGF-*β*1-induced+LMWF (200 *μ*g mL^−1^) (LMWF), TGF-*β*1-induced+LMWF (100 *μ*g mL^−1^), and TGF-*β*1-induced+LMWF (50 *μ*g mL^−1^) groups. The proteins in the A549 cells were quantified by Western blot analyses, as detailed in a previous study, and visualized by chemiluminescence, using *β*-actin as a loading control.

### 2.9. Statistical Analysis

The results of Masson staining, Sirius red staining, and immunohistochemistry staining were analyzed using ImageJ software, and statistical analyses were carried out using GraphPad Prism 8.0 software. The variations among groups were assessed by one-way analysis of variance (ANOVA), followed by Dunnett's test. All of the quantitative data are expressed as the mean ± SD, and differences were considered to be statistically significant and very significant at *p* < 0.05 (∗/#) and *p* < 0.01 (∗∗/##), respectively.

## 3. Results

### 3.1. Chemical Composition of LMWF

**Figure 1:**
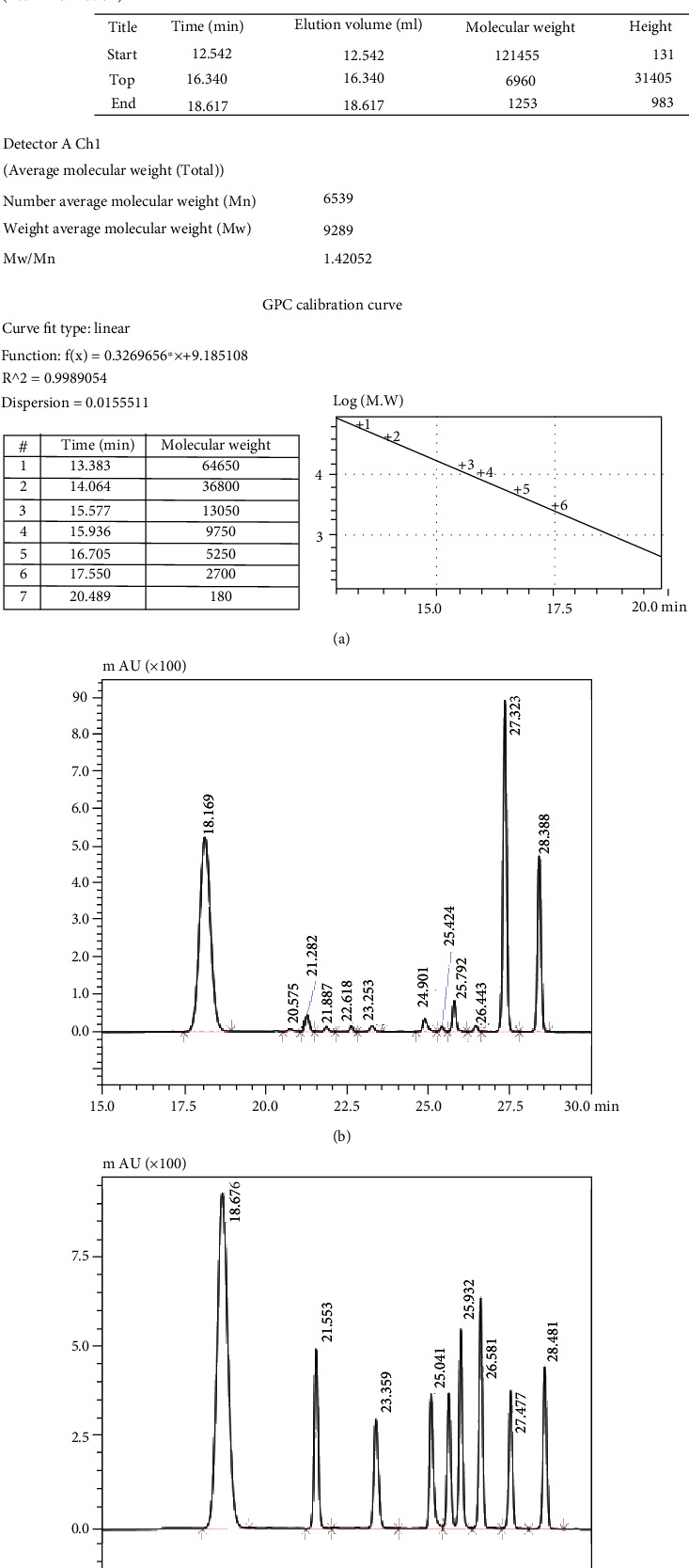
(a) Determination of LMWF molecular weight by high-performance liquid chromatography with gel permeation chromatography; (b) determination of the peak time of a neutral sugar standard by HPLC; (c) determination of the peak time of a neutral sugar in LMWF by HPLC; (d) chemical composition and neutral sugar composition of FPS; abbreviation: Fuc: fucose; Gal: galactose; Man: mannose; Glc: glucose; Rha: rhamnose; Xyl: xylose; Rib: ribose.

As shown in Figure 1(a), the molecular weight of LMWF was found to be 9289 Da (*Mw*/*Mn*: 1.42). Based on HPLC analysis after PMP precolumn derivatization, fucose is the main monosaccharide component of LMWF, followed by galactose and rhamnose (Figures 1(b) and 1(c)). Using the appropriate chemical methods, the total sugar, sulfate, and fucose contents were determined to be 63.16%, 28.14%, and 30.62%, respectively (Figure 1(d)).

### 3.2. Pathological Changes in Mouse Lung Tissues

**Figure 2:**
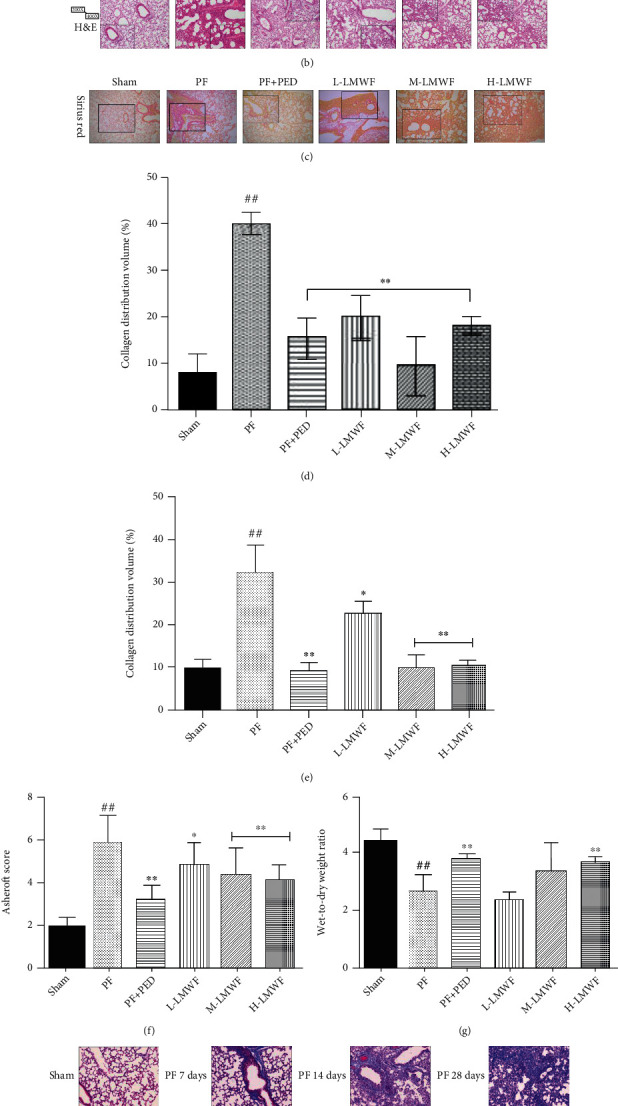
(a) Masson staining was used to observe pulmonary fibrosis in mice; (b) hematoxylin and eosin staining to observe lung injury in mice; (c) Sirius red staining to observe collagen deposition in the lung of mice; (d) collagen distribution volume statistics using Masson staining images (calculated by Image J); (e) collagen distribution volume statistics for Sirius red staining images (calculated by Image J) (^∗^*p* < 0.05 and ^∗∗^*p* < 0.01 vs. the PF group; ^##^*p* < 0.01 vs. the Sham group); (f, e) Ashcroft score and the wet-to-dry ratio for each group (^∗^*p* < 0.05 and ^∗∗^*p* < 0.01 vs. the PF group; ^##^*p* < 0.01 vs. the Sham group); (h) Masson staining of lung tissues from mice injected with bleomycin at different time points.

Based on Masson staining, there was obvious collagen deposition (blue part) in the lungs of PF mice. Compared to these mice, the PED and LMWF mice exhibit less collagen deposition. Moreover, the pathological changes observed in the LMWF group were similar to those detected in the PED group (Figure 2(a)).

The results of H&E staining demonstrated that the lungs of PF mice exhibited obvious intraluminal exudation with serious infiltration of perivascular inflammatory cells. In addition, the alveolar septa of these mice were smaller than those of Sham mice. Comparatively, the PED and LMWF (low-, medium-, and high-dose) groups showed significantly reduced lung inflammation and improved pathological condition, but the improvement was less than that observed for PED mice (Figure 2(b)).

The Sirius red staining images showed that the number of collagen fibers in the lung tissue of PF mice was significantly greater than the number detected in Sham mice. PED and LMWF treatments inhibited the number of PF-induced increase in collagen fibers. However, the effect in the low-dose group was less than that of the other two treatments (Figure 2(c)). Notably, the PED and high-dose LMWF groups exhibited similar numbers of collagen fibers as the Sham group. Statistical analysis of the Sirius red staining and Masson staining images (Figures 2(d) and 2(e)) shows that the collagen volume in the PF group was significantly greater than that in other groups (^##^*p* < 0.01 vs. Sham group), which indicated that medium-dose PED and high-dose LMWF had obvious PF inhibition effects (^∗^*p* < 0.05 and ^∗∗^*p* < 0.01 vs. PF group). Ashcroft scoring is presented in Figure 2(f). The PED and high-dose LMWF groups showed effective inhibition of PF. Lung wet-to-dry weight ratios (*W*/*D*) in each group are shown in Figure 2(g).

### 3.3. Expression and Localization of Fibrosis Markers

**Figure 3:**
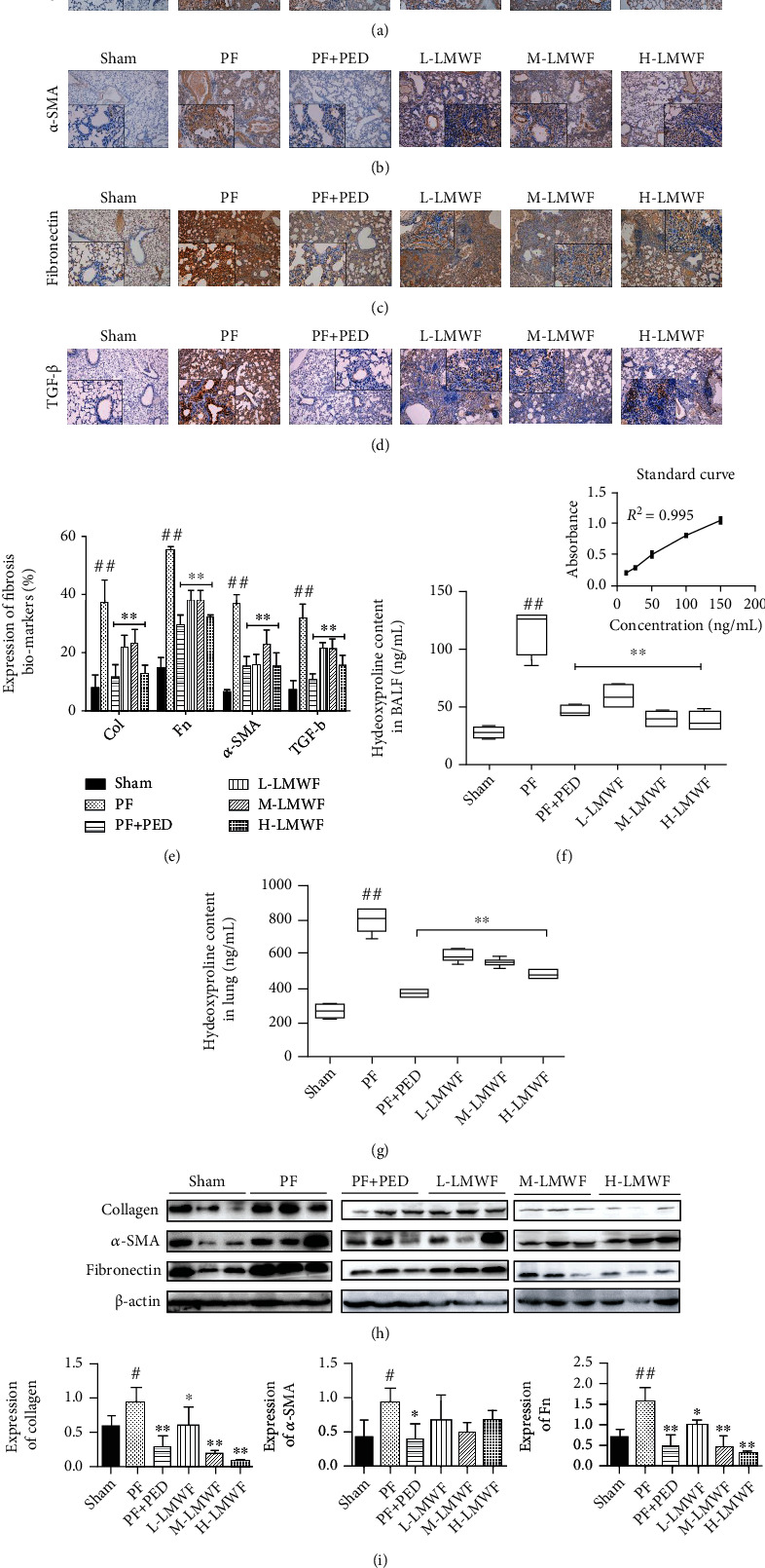
Assessment of pulmonary fibrosis. Immunohistochemical detection of fibrosis biomarkers: (a) collagen-1, (b) *α*-SMA, (c) fibronectin, and (d) TGF-*β*; (e) statistical expressions of fibrosis biomarkers by an immunohistochemical assay (^∗∗^*p* < 0.01 vs. the PF group; ^##^*p* < 0.01 vs. the Sham group); (f, g) hydroxyproline in the BALF and lung (^∗∗^*p* < 0.01 vs. the PF group; ^##^*p* < 0.01 vs. the Sham group); (h) expression of fibrosis biomarkers tested by Western blot; (i) statistical expressions of fibrosis biomarkers in by Western blot (^∗^*p* < 0.05 and ^∗∗^*p* < 0.01 vs. the PF group; ^#^*p* and ^##^*p* < 0.01 vs. the Sham group).

The expression of fibrosis markers was monitored using immunohistochemistry analyses. The obtained results showed that the deposition of collagen was significantly increased upon the onset of pulmonary fibrosis and that the different three doses of LMWF and PED decrease this deposition. High-dose LMWF further limited the effect of PF in increasing collagen deposition (Figure 3(a)). The trends of *α*-SMA and fibronectin expression were similar to that of collagen. Specifically, high-dose LMWF treatments significantly improved the changes in expression induced by PF (Figures 3(b) and 3(c)). Similarly, PF increased the expression of TGF-*β* in mouse lung tissue (Figure 3(d)), and treatment with LMWF or PED inhibited this increase.  Figure 3(e) depicts the statistical analysis of collagen, *α*-SMA, fibronectin, and TGF-*β*. Using ELISA, we found that PF increased the secretion of hydroxyproline compared to the Sham group, and treatment with LMWF or PED restricted this PF-induced increase (Figures 3(f) and 3(g), ^∗^*p* < 0.05 and ^∗∗^*p* < 0.01 vs. PF group).

To assess the expression of pulmonary fibrosis markers more accurately, the lung tissues of mice were ground, and the protein in these tissues was extracted and detected by Western blotting (Figure 3(h)). The obtained results demonstrated that PF significantly increased the expression of collagen and fibronectin. Treatment with LMWF or PED, however, countered this increase. Among the investigated groups, the PED and high-dose LMWF groups exhibited fibrosis marker expression similar to that of the Sham group (Figure 3(i)).

### 3.4. Oxidative Stress in the Lung

**Figure 4:**
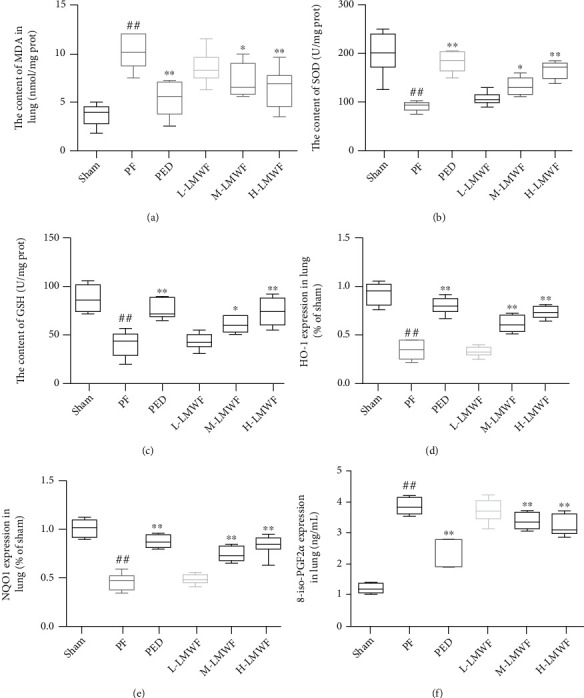
Assessment of oxidative stress in the lung: (a) MDA, (b) SOD, and (c) GSH content in each group; (e)HO-1, (f) NQO1, and (g) 8-ios-PGF2*α* by ELISA (^∗∗^*p* < 0.01 vs. the PF group; ^##^*p* < 0.01 vs. the Sham group).

Oxidative stress was assessed by measuring lung MDA and SOD levels in PF mouse. The content of MDA increased significantly in the PF group, but it decreased in the PED and LMWF groups. The MDA content change showed concentration dependence (Figure 4(a)). The change in SOD and GSH content was opposite to that of MDA, but it also showed a concentration dependence, as the high-dose LMWF group was significantly better (Figures 4(b) and 4(c)). The concentrations of HO-1 and NQO1 were detected by ELISA, and then, other groups were compared with the Sham group. The Sham group was used as the benchmark for proportional conversion. The results showed that the contents of HO-1 and NQO1 in the lungs of mice in the PF group decreased significantly, while the results from the PED and LMWF groups showed a more positive response after intervention (Figures 4(d) and 4(e)). As another important regulator of oxidative stress, the expression of 8-iso-PGF2*α* increased significantly in the lungs of mice with PF but decreased after being regulated by PED and LMWF, and the effect of high-dose LMWF was better than either of these (Figure 4(f)).

### 3.5. In Vitro Experiments

**Figure 5:**
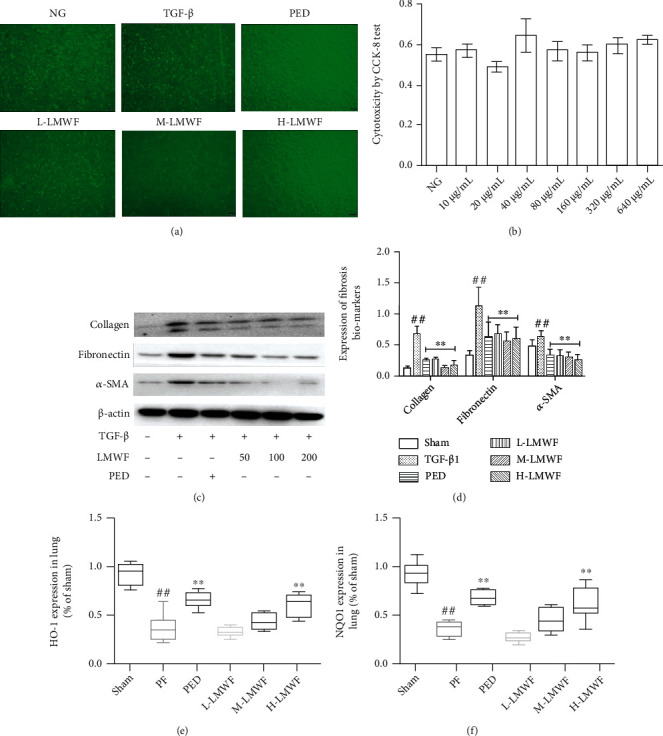
Assessment of pulmonary fibrosis in A549 cells. (a) Observing the state of A549 cells; (b) cytotoxicity of LMWF in A549 cells; (c) detection of fibrosis biomarker protein expression by Western blot; (d) statistical protein expressions of fibrosis biomarkers of pulmonary fibrosis (^∗^*p* < 0.05 and ^∗∗^*p* < 0.01 vs. the TGF-*β* group; ^#^*p* and ^##^*p* < 0.01 vs. the NG group); (e, f) statistical HO-1 and NQO1 expressions by ELISA (^∗^*p* < 0.05 and ^∗∗^*p* < 0.01 vs. the TGF-*β* group; ^#^*p* and ^##^*p* < 0.01 vs. the NG group).

*In vitro* experiments were used to assess the toxicity of LMWF in A549 cells. As shown in Figure 5(a), this polysaccharide is nontoxic in a concentration range from 0 to 640 *μ*g mL^−1^. Unlike TGF-*β*-induced cells, which exhibit obvious fibrosis symptoms and spindle shapes, the cells treated with 200 *μ*g mL^−1^ PED and a high dose of LMWF showed no appreciable changes in morphology compared to normal cells (Figure 5(b)). Western blots confirmed that TGF-*β* induced a significant increase in the expression of fibrotic markers (Figures 5(c) and 5(d)), as well as the expression of HO-1 and NQO1 (tested by ELISA, Figures 5(e) and 5(f)), which was consistent with our *in vivo* experimental results.

## 4. Discussion

In early studies, N-acetylcysteine was used in combination to inhibit PF, but it is no longer recommended because of its large toxic side effects [31]. Subsequent studies have shown that gastroesophageal reflux was one of the stimulating factors for the onset and deterioration of PF, and proton pump inhibitors have been proposed as anti-PF drugs [32]. However, long-term use of proton pump inhibitors has many adverse effects, such as infection, cognitive function problems, and myocardial infarction [33]. With the gradual deepening of PF pathological research, researchers began to believe that successful antifibrotic drugs can not only inhibit inflammatory response and reduce myofibroblast proliferation, but they can also block the imbalance of oxidation/antioxidant effects [34, 35]; however, there have been certain drug risks. Although considerable progress has been made in the treatment of PF and relevant medication guidance has been clinically formulated [36], there are still some problems, such as the dearth of available drugs and unclear safety of these drugs. Through this study, we found that LMWF could effectively inhibit the development of PF and provide more potential effective drugs for the treatment of PF. LMWF can effectively alleviate the drain of PF on public health resources.

Fucose is the main monosaccharide component of LMWF (highest content), followed by galactose. The relative proportion of fucose and sulfate groups was shown to be near 1 : 1, which was consistent with previous studies conducted on *Laminaria japonica* polysaccharides [37]. This proportion enhances the medicinal efficiency of LMWF, and it shows that the basic structure of fucoidan was not destroyed by free radical degradation.

**Figure 6:**
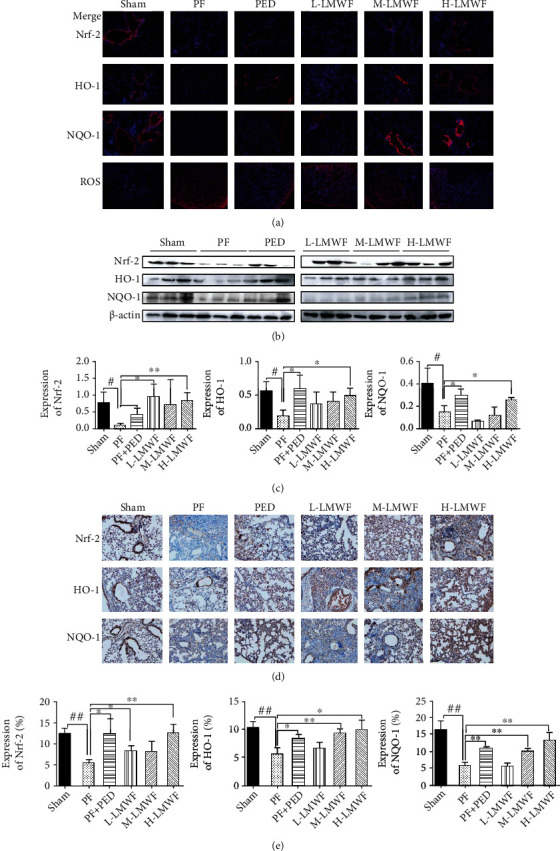
(a) Immunofluorescence detection of Nrf-2, HO-1, NQO1, and ROS; (b) expression of Nrf-2, HO-1, and NQO1 tested by Western blot; (c) statistical expressions of Nrf-2, HO-1, and NQO1 by Western blot (^∗^*p* < 0.05 vs. the PF group; ^#^*p* < 0.05 vs. the Sham group); (d) immunohistochemical detection of Nrf-2, HO-1, and NQO1; (e) statistical expressions of Nrf-2, HO-1, and NQO1 by an immunohistochemical assay (^∗^*p* < 0.05 and ^∗∗^*p* < 0.01 vs. the PF group; ^#^*p* < 0.05 and ^##^*p* < 0.01 vs. the Sham group).

Oxidative stress refers to a pathological state in which the body produces too many reactive nitrogen free radicals and reactive oxygen free radicals after some stimulation, resulting in an oxidation/antioxidant imbalance. Oxidative stress is the main cell stressor, and it can act on cells directly or indirectly, resulting in structural necrosis, apoptosis, and tissue inflammation. Excessive oxidants in oxidative stress environments will damage epithelial cells and further induce PF [38]. Antioxidant stress, then, is the key to successful PF treatment. Studies have confirmed that antioxidant enzyme proteins can prevent oxidative stress injury and reduce pulmonary fibrosis [39]. Nrf-2 is the most representative nuclear transcription factor regulating oxidative stress [11]. When stimulated by oxidants, Nrf-2 is released, translocates into the nucleus, and combines with antioxidant response elements to induce the transcription of downstream SOD, MDA, GSH, and other antioxidant enzyme genes to regulate the expression of antioxidant enzymes in cells. At the same time, Nrf2 regulates OS by activating downstream antioxidant proteins, including heme oxygenase (HO-1) and NAD(P)H: quinone oxidoreductase (NQO1) [40]. In this study, we found that the lung oxidative stress of PF mice was significantly increased, and the test results of oxidative stress-related regulatory enzymes (SOD, MDA, and GSH) in mouse lung tissue showed that pulmonary fibrosis resulted in significant enhancement of oxidative stress and an imbalance in antioxidant capacity, but this oxidative stress was regulated after LMWF intervention. This showed that LMWF could effectively reduce the oxidative pressure of lung tissue in PF. Moreover, there was a dose dependence, with high-dose LMWF displaying better effects. In order to clarify the mechanism of LMWF regulating pulmonary fibrosis in mice through antioxidant effects, we studied the expression of Nrf-2 and its regulated HO-1, NQO1, and ROS proteins by immunofluorescence, Western blotting, and immunohistochemistry (Figures 6(a)–6(c)). Through immunofluorescence detection (blue is the nucleus, and red is the target protein), we found that the expression of Nrf-2 in the lung tissue of PF mice decreased significantly, and after intervention with high-dose LMWF, the lung antioxidant capacity of PF mice improved. Then, the same detection was carried out for HO-1 and NQO1 regulated by Nrf-2, and the observation results were similar to the expression of Nrf-2. Since immunofluorescence can only observe the spatial distribution of a target protein but cannot be used to carry out quantitative analysis, we then carried out semiquantitative analysis using Western blotting and immunohistochemistry. Western blotting and immunohistochemistry results demonstrated that the expression of Nrf-2 and its regulated HO-1 and NQO1 were significantly downregulated in the lung tissue of PF mice (Figures 6(d) and 6(e)). Combined with the previous results of pulmonary oxidative stress tests, we found that LMWF inhibited fibrosis by regulating the expression of Nrf-2, HO-1, and NQO1 through its antioxidant activity. Nrf-2 is an important factor in antioxidant regulation *in vivo*. The results also showed that the expression of Nrf-2 was significantly increased in the lung tissue of PF mice, and LMWF effectively regulated this.

In addition to these *in vivo* experiments, the mechanism of PF inhibition by LMWF was also examined *in vitro*. Specifically, TGF-*β*1 was used to induce fibrosis in A549 cells. At 72 hours after induction, cells showed obvious morphological changes. However, LMWF and PED intervention significantly improved the morphology of cells. Moreover, LMWF and PED could effectively reduce the expression of fibrosis marker proteins. Considering that LMWF can also regulate the expression of HO-1 and NQO1, it may be concluded that oxidative stress is implicated in the activity of LMWF against PF. The *in vivo* and *in vitro* experiments conducted in this study indicated that LMWF inhibits PF via antioxidant activity.

## Data Availability

If necessary, readers can contact the corresponding author by email to provide the original data.
