# Correction to “Low Molecular Weight Fucoidan Inhibits Pulmonary Fibrosis In Vivo and In Vitro via Antioxidant Activity”

**DOI:** 10.1155/omcl/9854582

**Published:** 2025-10-23

H. Dong, T. Xue, Y. Liu, et al., “Low Molecular Weight Fucoidan Inhibits Pulmonary Fibrosis In Vivo and In Vitro via Antioxidant Activity,” *Oxidative Medicine and Cellular Longevity* 2022 (2022): 7038834, https://doi.org/10.1155/2022/7038834.

In the article, an error was introduced during the production process in Figure 1b. Specifically, the H-LMWF panel was erroneously duplicated in the M-LMWF panel. The correct Figure 1 is shown below:

We apologize for this error.

## Figures and Tables

**Figure 1:**
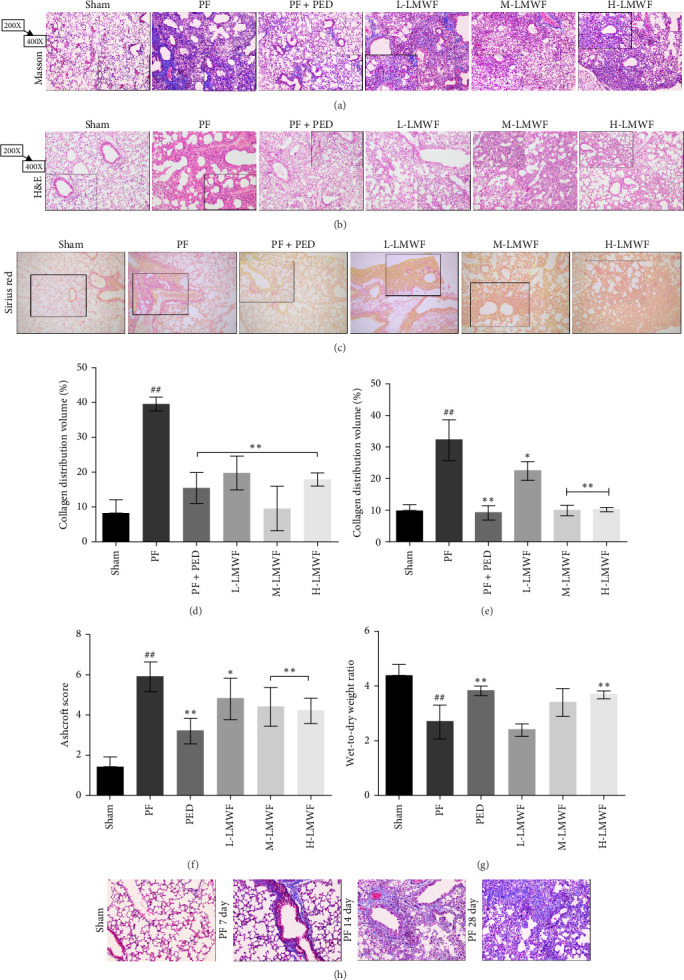
(a) Masson staining was used to observe pulmonary fibrosis in mice; (b) hematoxylin and eosin staining to observe lung injury in mice; (c) Sirius red staining to observe collagen deposition in the lung of mice; (d) collagen distribution volume statistics using Masson staining images (calculated by Image J); (e) collagen distribution volume statistics for Sirius red staining images (calculated by Image J) (*⁣*^*∗*^*p* < 0.05 and *⁣*^*∗∗*^*p* < 0.01 vs. the PF group; ^##^*p* < 0.01 vs. the Sham group); (f, g) Ashcroft score and the wet-to-dry ratio for each group (*⁣*^*∗*^*p* < 0.05 and *⁣*^*∗∗*^*p* < 0.01 vs. the PF group; ^##^*p* < 0.01 vs. the Sham group); (h) Masson staining of lung tissues from mice injected with bleomycin at different time points.

